# Gene-based therapies for neuromuscular disorders

**DOI:** 10.1055/s-0043-1777755

**Published:** 2024-02-07

**Authors:** Edmar Zanoteli, Marcondes Cavalcante França, Wilson Marques

**Affiliations:** 1 Universidade de São Paulo, Faculdade de Medicina, Departamento de Neurologia, São Paulo SP, Brazil.; 2 Universidade Estadual de Campinas, Faculdade de Ciências Médicas, Departamento de Neurologia, Campinas SP, Brazil.; 3 Universidade de São Paulo, Faculdade de Medicina de Ribeirão Preto, Departamento de Neurociências e Ciências do Comportamento, Ribeirão Preto SP, Brazil.

**Keywords:** Gene Therapy, Muscular Atrophy, Spinal, Amyotrophic Lateral Sclerosis, Amyloid Neuropathies, Muscular Dystrophy, Duchenne, Terapia Genética, Atrofia Muscular Espinhal, Esclerose Amiotrófica Lateral, Neuropatias Amiloides, Distrofia Muscular de Duchenne

## Abstract

Neuromuscular diseases (NMD) include a broad group of medical conditions with both acquired and genetic causes. In recent years, important advances have been made in the treatment of genetically caused NMD, and most of these advances are due to the implementation of therapies aimed at gene regulation. Among these therapies, gene replacement, small interfering RNA (siRNA), and antisense antinucleotides are the most promising approaches. More importantly, some of these therapies have already gained regulatory approval or are in the final stages of approval. The review focuses on motor neuron diseases, neuropathies, and Duchenne muscular dystrophy, summarizing the most recent developments in gene-based therapies for these conditions.

## Resumo

Doenças neuromusculares (DNM) compõem um grupo amplo de doenças de causa tanto adquiridas quanto genéticas. Nos últimos anos, importantes avanços ocorreram quanto ao tratamento das DNM de causa genética e grande parte desses avanços se deve à implementação de terapias voltadas para a modificação gênica. Dentre essas terapias, destacam-se as terapias de reposição gênica, uso de RNA de interferência, uso de antinucleotídeos *antisense* , entre outras. E, mais importante, algumas dessas terapias já se tornaram realidade na prática médica e já foram aprovadas, ou estão a poucos passos da aprovação, por órgãos governamentais regulatórios. Esta revisão aborda aspectos mais recentes quanto ao uso das terapias genéticas avançadas para algumas das formas mais comuns de DNM, em especial para doenças do neurônio motor (esclerose lateral amiotrófica e atrofia muscular espinhal), neuropatias e distrofia muscular de Duchenne.

## INTRODUCTION

We have witnessed a real revolution in the therapeutic landscape of neuromuscular disorders (NMD) in the past 5–10 years. 1 This is particularly evident for monogenic conditions, where precise disease targets and mechanisms are well known. Diseases such as spinal muscular atrophy and hereditary transthyretin amyloidosis (ATTRv) are now treated effectively with multiple approved disease-modifying agents. 2 3 4 5 6 These innovative drugs in NMD are called gene-based therapies and rely upon the modulation of genes, mRNA, and/or proteins. 7 There are three main classes of such therapies: antisense oligonucleotides (ASOs), small interfering RNAs (RNAi), and gene replacement therapy (GT).

ASOs are synthetic single-stranded nucleic acid sequences containing between 8 and 20 nucleotides, which can bind to specific RNA sequences and thus regulate gene expression. Such ASO-mediated gene regulation can be mediated by activation of RNAse-H, by blocking mRNA transcription at ribosomes, or by modifying gene splicing. 8

RNAi-based drugs are small non-coding double-stranded RNA molecules with 21 to 23 nucleotides. These agents are designed to target specific mRNAs, and cause gene silencing through a complex multienzymatic intracellular system (RNA-induced silencing complex). 9 So far, RNAi-therapies have primarily been used intravenous (IV) or subcutaneously to treat autosomal dominant gain-of-function NMD (ATTRv and porphyrias).

GT consists of the insertion of genes into an individual's cells or tissues with the aim of treating or preventing a hereditary disease. There are two key components in all GT: the vector (which can be viral or non-viral) and the transgene (which includes the coding sequence of the gene of interest plus regulatory regions). 10 The choice of the transgene and the design of the transgene will depend on the condition treated. For NMDs, this is typically a single shot injection given in vivo either IV or intrathecal (IT).

## GENE-BASED THERAPIES FOR AMYOTROPHIC LATERAL SCLEROSIS (ALS)

Amyotrophic lateral sclerosis (ALS) is the prototypical motor neuron disease in adults. The disease is characterized by degeneration of both upper and lower motor neurons, leading to progressive muscle weakness and atrophy. 11 12 Patients typically die 3 to 5 years after the onset of symptoms due to respiratory failure. Most patients with ALS (90%) have sporadic disease (sALS) with no known family history, whereas the remaining 5-10% are called familial ALS (fALS) because the disease segregates in other affected relatives. The genetic basis of ALS is now well characterized. fALS is considered a monogenic disorder with more than 20 distinct genes already associated. In contrast, monogenic sALS is rare; most patients indeed behave as oligo or polygenic conditions. 13 Most monogenic ALS subtypes segregate as autosomal dominant traits and are caused by gain-of-function missense variants (leading to abnormal protein folding and aggregation). 12 13

Advanced gene-based therapies are coming to the clinical arena of ALS. 14 15 Essentially all drugs in development target one of the genes known to be associated with fALS. *SOD1* was the first gene identified in fALS back in 1993. 16 For this reason, it is the leading gene when it comes to clinical drug development. Other genes such as *C9orf72* , *FUS* , and *ATXN2* , have been also explored lately. Further detail will be given below.

### SOD1-ALS

Tofersen is an antisense oligonucleotide (ASO) administered intrathecally every month that targets both wild-type and mutant *SOD1* alleles. 14 It was tested in a phase 3 clinical trial that recruited 72 patients in a 2:1 ratio treatment versus placebo. They were followed over 24 weeks using the decline in the Amyotrophic Lateral Sclerosis Functional Rating Scale (ALSFRS) score which was the primary efficacy measure. In this time frame, the drug resulted in a significant decline in the cerebrospinal fluid concentrations of SOD1 and in the plasma concentration of neurofilament light chains, a marker of axonal degeneration. Furthermore, severe drug-related side effects were rather infrequent (7%). Despite that, the study did not meet its primary efficacy endpoint in the placebo-controlled phase. Long follow-up data from the open-label extension phase, however, found a clear positive trend towards the treatment. These results in combination enabled regulatory approval for tofersen to treat patients with SOD1-ALS in the US in 2023. 17 There is an ongoing study focusing on presymptomatic carriers of *SOD1* mutations treated with tofersen and followed in the long term. 18

GT has been also explored in SOD1-ALS. In 2020, Mueller et reported two patients with SOD1-ALS and fast progression who underwent GT. 15 The investigational product was administered IT. AAVrh10 was the vector, and the transgene encoded a microRNA targeting *SOD1* mRNA. The first treated patient did not receive prior immunosuppression, so he developed severe meningoradiculitis, and no obvious change in the progression was noticed. Post-mortem spinal cord samples from this patient indeed confirmed *SOD1* silencing. In contrast, the second patient received IV steroids pre-GT, and the course was strikingly different remaining stable over 70 weeks (ALSFRS scores). These are encouraging results and other groups are now pursuing a similar approach to treat this monogenic subtype of ALS. 19

### Other genes ( *C9orf72* , *FUS* , and *ATXN2* )

*C9orf72* is the most frequent genetic cause of fALS in Caucasians. 11 A phase 1 clinical trial using an IT ASO (BIIB078) targeting this gene was conducted. 20 The authors recruited and followed 106 patients who were randomized in a 3:1 ratio to treatment versus placebo. Unfortunately, no significant change was noticed between the treatment and placebo groups, which led to the discontinuation of drug development. 20

FUS causes a very aggressive and early-onset, sometimes juvenile, subtype of ALS. 11 Jacifusen is a *FUS* -targeting ASO-administered IT that is currently under investigation at Columbia University. Some patients received the drug from 2019 to 2020 after FDA approval. Clinical benefit was not clear at this point, but post-mortem samples of these individuals confirmed the reduction of FUS expression in the spinal cord. A phase 3 clinical trial named FUSION is currently ongoing to assess the safety and efficacy profile of jacifusen in 64 patients with FUS-ALS over 29 weeks. 21

Intermediate CAG expansions at *ATXN2* have been associated with ALS in multiple populations. 22 In contrast to other previously mentioned genes, this is not a causative gene, but rather a risk gene. On the other hand, its frequency is much higher in the overall ALS public compared to the other monogenic ALS subtypes. In the transgenic TDP43 ALS murine model, anti-ATXN2 ASOs rescued the phenotype and prolonged survival, particularly when given early. 23 A phase 1 clinical trial is ongoing to assess the safety and efficacy of IT anti-ATXN2 ASOs in patients with sporadic and ATXN2-related ALS. 21

## SPINAL MUSCULAR ATROPHY LINKED TO 5q (SMA-5q)

Spinal muscular atrophy (SMA) is a neurodegenerative disease characterized by progressive muscle weakness, hypotonia, and weakness due to degeneration of motor neurons in the spinal cord and brainstem. The most common form of SMA is caused by recessive mutations in the survival motor neuron 1 ( *SMN1* ) gene located at 5q13 (SMA-5q). 24 The global incidence of SMA is estimated at 1 in 10,000 live births. 25

SMA has been classified into at least four subtypes depending on the patient's age at the disease's onset and the achievement of motor milestones. 26 In SMA type 1, or infantile form, children do not acquire the ability to sit, and the disease manifests at 0 to 6 months of age. In SMA type 2, clinical manifestations start between 6 and 18 months, and children are unable to walk unassisted. Children with SMA type 3 manifest the disease in the second year of life or later and can walk unaided. Finally, SMA type 4 is the adult form of the disease with the manifestations starting usually after the age of 18.

Exon 7 of the *SMN1* is not detectable in approximately 96% of SMA-5q patients, and approximately 4% of patients have a combination of the deletion and an intragenic mutation in the second allele. 24 27 *SMN2* is a centromeric copy of *SMN1* that does not provide the transcription of stable SMN protein due to the lack of exon 7 in most transcripts. 24 Several studies have demonstrated a strong inverse correlation between the number of *SMN2* copies and SMA severity. 24 28

Various therapeutic approaches are currently being developed for SMA. SMN-dependent therapies focus on addressing the SMN protein deficiency, such as gene therapy with *SNM1* gene replacement (onasemnogene abeparvovec-AVXS101), and inclusion of exon 7 in *SMN2* (nusinersen, risdiplam). 29 These therapies have already been approved by the leading international regulatory agencies and the Brazilian National Surveillance Agency (ANVISA).

Nusinersen (Spinraza®) is an antisense oligonucleotide (ASO) that targets an intronic splicing silencer site within the *SMN2* pre-messenger RNA downstream of exon 7. 30 31 This targeting of the splicing silencer allows for increased inclusion of exon 7 during mRNA processing, producing more functional SMN protein from the *SMN2* gene. As ASOs do not cross the blood-brain barrier, nusinersen must be administered via the intrathecal route.

The ENDEAR, a placebo-randomized clinical trial, demonstrated that nusinersen-treated SMA type 1 patients had more prolonged survival and significant improvements in motor function compared to those without treatment. 3 The CHERISH trial was a placebo-randomized study that enrolled 126 patients with late-onset SMA aged 2 to 12 years. 32 The study demonstrated that patients who received Nusinersen experienced clinically significant improvements in motor function compared to the control group.

The follow-up of approximately three years of patients with SMA types 2 and 3 treated with nusinersen showed improvements in motor function and stabilization of disease activity. 33 For SMA type 1, a follow-up of approximately 36.2 months showed a durable clinical response in a significant proportion of patients; 75% of the participants were still alive at the time of study closure. 34

Several subsequent real-life studies with SMA patients further confirmed the favorable effects of nusinersen on motor and respiratory function, as well as on the survival of patients with long-term illness and varying respiratory conditions. 35 36 37 38

NURTURE is an ongoing phase 2, open-label study to evaluate the efficacy and safety of nusinersen in pre-symptomatic infants. 39 With a median follow-up of 2.9 years, the infants (median of 34.8 months of age) had surpassed the expected age of symptom onset for SMA types 1 or 2, and all of them were alive without the need for tracheostomy or permanent ventilation. Almost all the participants achieved the ability to sit without support (92%), and the majority achieved walking with assistance or independently (88%). 39 After five years of follow-up, all patients were alive, and none discontinued the treatment or utilized respiratory intervention. Children with three *SMN2* copies achieved all WHO motor milestones, and all children with two *SMN2* copies achieved sitting without support, 4/15 walking with assistance, and 13/15 walking alone. 40

Risdiplam (Evrysdi®) is a small oral molecule designed to selectively modify the splicing of *SMN2* pre-mRNA and promote the inclusion of exon 7 to increase levels of functional SMN protein from a complete mRNA transcript. 41 Registration approval in Brazil occurred in October 2020.

The FIREFISH study is investigating the safety and efficacy of risdiplam in type 1 SMA versus historical controls. 4 42 After 24 months of treatment, 44% of the patients were sitting without support for at least 30 seconds. 43 The event-free survival at month 12 was 85% and at month 24 was 83%. 42 44 The most frequently reported adverse event was upper respiratory tract infection in 54%. 44 SUNFISH (NCT02908685), a phase 3, randomized, placebo-controlled study, investigates the effects of risdiplam in type 2 and non-ambulant type 3 SMA. 45 Part 1 of the study showed that a median two-fold increase of serum SMN protein was obtained within four weeks of treatment, and it was sustained over 24 months of treatment. 45 In part 2 of the study, an exploratory efficacy showed improved or stabilized motor function. A significantly greater change from baseline in the 32-item Motor Function Measure (MFM32) total score was observed with risdiplam compared with placebo at month 12. 46 At month 24, 32% of patients demonstrated improvement from baseline in MFM32 total score, and 58% showed stabilization. 47

The JEWELFISH is an ongoing, open-label study designed to assess the effects of risdiplam in the broadest population, including patients with SMA types 1–3 (n = 174) with a wide range of ages (1–60 years), disease severities, and who have previously received other therapies (RG7800, 7 nusinersen, olesoxime or onasemnogene abeparvovec). 48 The study showed a favorable safety profile and an increase in SMN protein levels after 12 months of treatment. 48 An increase in the total distance walked in the 6MWT was observed in ambulant patients over 24 months of treatment with risdiplam. 48 Real-world experience with risdiplam has also been published and further supported its beneficial effects on motor function in patients with SMA. 49

RAINBOWFISH is an ongoing, multicenter, open-label, single-arm study to assess the efficacy and safety of risdiplam in pre-symptomatic SMA. Preliminary data showed that most infants treated with risdiplam could sit independently, and many were standing and walking as assessed by the HINE-2 at month 12. 43 After 12 months of treatment, most infants achieved near-maximum CHOP-INTEND total scores. All infants maintained bulbar function, and none required permanent ventilation after 12 months of treatment. 43

Onasemnogene abeparvovec (Zolgensma®) is a gene replacement therapy based on a self-complementary adeno-associated virus serotype 9 (AAV9) vector that carries a functional copy of the human *SMN1* . 2 The administration of onasemnogene abeparvovec is performed intravenously, allowing the AAV9 vector to cross the blood-brain barrier. In the US, the FDA has approved it for the treatment of children with SMA who are under two years of age. In Brazil, the ANVISA has approved onasemnogene abeparvovec for babies younger than two years old.

The START study was a pivotal clinical trial that evaluated the safety and efficacy of onasemnogene abeparvovec in patients with type 1 SMA who had two copies of the *SMN2* . At 20 months following the treatment, 11 of the 12 children receiving the high dose of gene therapy could sit unassisted and feed unassisted. 2 Data from the extension study showed maintenance of the effectiveness for at least five years. 50

The phase 3 studies STR1VE-EU conducted in Europe and STR1VE-US conducted in the US further confirmed the effectiveness of onasemnogene abeparvovec in treating patients with type 1 SMA when administered before six months of age. 51 52 Between 44% to 59% of treated patients could sit unsupported at 18 months. Furthermore, between 91% to 97% of the infants were alive and free from mechanical ventilation at 14 months of age. 51 52

Real-world studies have confirmed the efficacy of the gene replacement therapy in an expanded age range of patients eligible for treatment, including those up to two years old, and also patients with three copies of *SMN2* , regardless of the type of SMA. 53 54 55 Additionally, some studies have evaluated the use of onasemnogene abeparvovec in patients who had previously been treated with other specific therapies, such as nusinersen or Risdiplam. 53 54

The SPR1NT phase 3 study has provided crucial evidence on the efficacy of gene replacement therapy in pre-symptomatic children with SMA. 56 57 In children with three *SMN2* copies, all 15 participants stood independently before 24 months, within the expected developmental window. 57 Additionally, 14 of them walked independently within the expected developmental window, and 67% maintained body weight without requiring feeding support through 24 months. For the 14 enrolled infants with two *SMN2* copies, all of them achieved the ability to sit independently for ≥30 seconds at any visit before 18 months of age. 56 Importantly, all patients with two or three *SMN2* copies in the study survived without permanent ventilation at 14 months, and none of the children required nutritional or respiratory support. At 18 months (children with two copies of *SMN2* ) and 24 months (children with three copies of *SMN2* ), all children swallowed normally and achieved full oral nutrition. 58

In human clinical trials and real-world studies, treatment-related severe adverse events of onasemnogene abeparvovec have been reported in just over 10% of cases, with the most common being liver function abnormalities and fever. 59 Other important adverse events include decreased platelet count and thrombocytopenia. Fatal cases of thrombotic microangiopathy and acute liver failure have been reported, 60 61 as well as potentially fatal conditions such as hemophagocytic syndrome, 62 and necrotizing enterocolitis. 63

The STRONG study (NCT03381729) is a clinical trial that evaluated the safety and efficacy of an intrathecal single dose of onasemnogene abeparvovec in non-ambulatory patients with SMA who have three copies of the *SMN2* and are aged 6 to under 60 months. 64 In the younger group (6 to under 24 months) treated with the medium dose, one out of thirteen patients (7.7%) achieved independent standing. For the older group (24 to under 60 months) receiving the medium dose, there was a significant improvement in the change from baseline in the HFMSE compared with the SMA historic control population at month 12. 64 Further research is ongoing to explore the use of lower intrathecal doses of onasemnogene abeparvovec in patients aged 2 to under 18 years in a randomized multicenter controlled clinical trial (NCT05089656).

## GENE-BASED THERAPIES FOR NEUROPATHIES

Among the inherited peripheral neuropathies, outstanding treatment developments occurred in the last decade. The natural history of patients with transthyretin-associated amyloidosis and acute hepatic porphyria with repetitive crisis has definitely changed due to genetic therapies.

### Hereditary transthyretin amyloidosis

Mutations in the transthyretin gene ( *TTR* ) result in an amyloidogenic multisystemic disease (ATTRv) that affects mainly the peripheral nervous system (ATTRv-pn) and the heart (ATTRv-h). 65 66 Most of the TTR protein has liver production and circulates as a monotetramer, that transports thyroxin and vitamin A. 65 66

ATTRv is a fatal autosomal dominant disease of variable penetrance caused by the deposition of misfolded TTR fibrils whose prognosis changed enormously after liver transplantation. The natural history of the disease was also modified with the introduction of tafamidis, a small molecule that stabilizes the TTR tetramer, avoiding fibril formation. 65 66

The possibility of interrupting TTR production through gene silencing became a clinical reality in the second half of the last decade with the use of two different technologies: antisense oligonucleotides (ASO) and small interfering RNAs (siRNA), both effectively suppressing TTR production acting at RNA level. 66 ASO selectively binds to the complementary RNA, preventing RNA translation and gene expression, which can also be modulated by siRNA which targets and cleaves complementary mRNAs. 67

The NEURO-TTR trial showed that the antisense oligonucleotide inotersen was effective in stages 1 and 2 of the ATTRv-pn. 5 Administered subcutaneously weekly it was well tolerated and effective in stabilizing the disease, but three patients developed glomerulonephritis, and the other three developed thrombocytopenia, with one death. After the introduction of regular monitoring, no more serious complications were reported. The open-label extension study confirmed the efficacy of inotersen in slowing the course of neuropathy and improving quality of life. 68

More recently, in trying to improve the safety and dosing profile of inotersen, the same sequence has been ligand-conjugated to produce a new and potent ASO (eplontersen), which is administered subcutaneously every 4 weeks. 69 70 The phase 3 NEURO-TTRansform study showed that the eplontersen treatment group had significantly lowered serum transthyretin concentration, less neuropathy impairment, and better quality of life compared with a historical placebo. 69

The phase 3 APOLLO study showed that the siRNA patisiran was effective and safe at the dose of 0.3 mg/kg given intravenously every 3 weeks. 6 The open-label extension study confirmed the efficacy and safety of the drug, 71 and it was shown that patisiran was also efficient in transplanted patients. 72 In phase 3 (APOLLO-B, NCT03997383), a double-blind, placebo-controlled, randomized trial, the administration of patisiran over a period of 12 months resulted in preserved functional capacity in patients with ATTR cardiac amyloidosis. 73 Infusion-related reactions, arthralgia, and muscle spasms occurred more often among patients in the patisiran group than among those in the placebo group. 73

The HELIOS-A study showed that vutrisiran, a siRNA-GalNAc conjugate, administered subcutaneously (25 mg every 3 months), resulted in significant improvement of the neuropathy impairment. 74

Gene editing therapy with the use of clustered, regularly interspaced short palindromic repeats and associated Cas9 endonuclease is also under evaluation to potentially solve the problem permanently. In the initial proof-of-concept study, single doses (0.3 mg/kg) of the NTLA-2001 CRISPR-Cas9 system resulted in a significant reduction of TTR blood levels without significant adverse events. 75 Ongoing and larger studies will test the clinical efficacy and safety of this system. 75

### Acute hepatic porphyria

Acute hepatic porphyria is a group of four diseases, three of autosomal dominant inheritance (acute intermittent porphyria, hereditary coproporphyria, variegate porphyria) and one autosomal recessive (ALA dehydratase) that result from abnormalities in the heme biosynthesis pathway. The penetrance is reduced and most carriers will never manifest the disease, but in those that manifest, the consequences may be serious and even fatal. 76 They usually occur in crises that cause intense abdominal pain, nausea, vomiting, dysautonomia (tachycardia and blood pressure instability), psychiatric problems, seizures, and acute axonal neuropathy. 77 Most patients have favorable prognoses with measures to avoid the precipitating factors and use of glucose and hematin at the onset of the attacks.

For those developing repetitive crises (> 4 a year) it has recently been introduced givosiran, a siRNA that binds to a target sequence on ALAS1 mRNA, decreasing the production of ALA and PBG, the molecules thought to be responsible for the porphyria attacks. The phase 3 ENVISION trial showed a 74% decrease in the attack rate, and the secondary endpoints were also met. 78 Givosiran was well tolerated, but some patients developed renal and hepatic complications, demanding close surveillance. 77 The recommended dose is 2.5 mg/kg once a month. Extension studies are ongoing.

## GENE-BASED THERAPIES FOR DUCHENNE MUSCULAR DYSTROPHY

Duchenne muscular dystrophy (DMD) is an X-linked recessive muscular dystrophy caused by mutations in the *DMD* (dystrophin) gene. 79 The disease affects around 1 in 3500 - 5000 boys and causes progressive weakness starting between 3 and 4 years old and loss of ambulation at the age of around 10–12 years. 80 In addition, DMD patients present respiratory difficulties and progressive dilated cardiomyopathy leading to heart failure and early death.

The *DMD* is the largest gene identified with 79 exons. 81 *DMD* mutations are intragenic deletions affecting one or more exons (60-65%), duplications of one or more exons (10%), and nucleotide variants (around 20-25%), including missense and nonsense variants, small insertions/deletions, or splicing alterations. 82 Mutations in *DMD* that do not disrupt the reading frame, leading to the expression of a truncated yet functional dystrophin cause the milder phenotype of Becker muscular dystrophy. 83

There is no curative treatment for DMD. Nonetheless, a multidisciplinary approach targeting the symptoms of DMD can change the natural course of the disease. Glucocorticosteroids, such as deflazacort or prednisone, are the current standard of care treatment for DMD patients (Araujo 2023). 84 Genetic therapies that act by regulating the expression of *DMD* or administering a transcript capable of encoding a smaller but still functional dystrophin have been the most promising therapies.

### Gene replacement therapy with microdystrophin

The large size of the *DMD* gene does not allow its transfer via an AAV vector. On the other hand, microdystrophin transcripts can be easily transferred to DMD patients via viral vectors. 85 Delandistrogene moxeparvovec is approved in the US for the treatment of ambulatory patients (4-5 years) with DMD. Delandistrogene moxeparvovec (SRP-9001), from Sarepta Therapeutics, is an investigational gene therapy designed for targeted expression of SRP-9001 dystrophin protein, a shortened dystrophin retaining key functional domains of the wild-type protein. An open-label, phase 1/2a, nonrandomized controlled clinical trial (NCT03375164) enrolled four DMD ambulatory males with a mean age of 5.1 years. 86 Patients received a single IV infusion (2.0 × 1014 vg/kg) of delandistrogene moxeparvovec and steroids. 86 At 12 weeks, immunohistochemistry of gastrocnemius muscle specimens revealed robust transgene expression in all patients, with a mean of 81.2% of muscle fibers expressing micro-dystrophin with a mean intensity of 96% at the sarcolemma. Western blot showed a mean expression of 74.3% of the protein without fat or fibrosis adjustment. 86 After 4 years of treatment, there were 18 treatment-related adverse events; all occurred within 70 days posttreatment and were resolved. 87 The mean North Star Ambulatory Assessment (NSAA) total score increased from 20.5 to 27.5 (+7.0, 2.9), from baseline to year 4.

SRP-9001-102 (NCT03769116) is a phase 2, double-blind, two-part crossover study to evaluate delandistrogene moxeparvovec in DMD patients aged ≥4 to <8 years. 88 Patients were randomized and stratified by age to placebo (n = 21) or delandistrogene moxeparvovec (n = 20) and crossed over for part 2. SRP-9001 dystrophin expression was achieved in all patients: mean change from the baseline to week 12 was 23.82% and 39.64% in parts 1 and 2, respectively. 88 In part 1, the change from the baseline to week 48 in NSAA score was +1.7 for treatment versus +0.9 for placebo. 88 In 4- to 5-year-olds with matched baseline motor function, the change from the baseline to week 48 in NSAA scores was significantly different (+2.5 points), but not significantly different in 6- to 7-year-olds with imbalanced baseline motor function (-0.7 points). For patients treated with delandistrogene moxeparvovec in part 2, the change from the baseline to week 48 in NSAA score was +1.3, whereas, for those treated in part 1, NSAA scores were maintained. 88 The most common adverse events were vomiting, decreased appetite, and nausea. 88

ENDEAVOR (NCT04626674) is a single-arm, open-label study to evaluate delandistrogene moxeparvovec in DMD boys. 89 In cohort 1 (n = 20), eligible ambulatory males, aged ≥4 to <8 years, received a single IV infusion of delandistrogene moxeparvovec (1.33 × 1014 vg/kg). At week 12, microdystrophin expression had a mean change from the baseline of 54.2% with sarcolemmal localization. 89 At one year, patients stabilized or improved in NSAA total scores. 89 Treatment versus a propensity score-weighted external natural history control demonstrated a statistically significant difference in NSAA (+3.2 points). 89 These positive results advanced SRP-9001 into a double-blind, placebo-controlled, phase 3 clinical trial called EMBARK (NCT05096221).

Fordadistrogene movaparvovec (PF-06939926), from Pfizer, is an AAV9 gene-replacement construct containing a truncated dystrophin transgene. A phase 1b open-label clinical trial (NCT03362502) has been testing the PF-06939926 in males with DMD aged 4 and older. 90 Nineteen ambulatory boys received fordadistrogene movaparvovec (n = 3 low-dose; n = 16 high-dose). Median age at gene therapy was 8.8 years (6.2–13.0 years). 90 Three treatment-related serious adverse events occurred (dehydration, acute kidney injury, thrombocytopenia); all resolved within 15 days. The median change from baseline to 1-year in NSAA total score was +1 point against -4 points for an external control cohort. 90 A randomized, placebo-controlled phase 3 trial, called CIFFREO (NCT04281485), is planned to enroll 99 DMD boys aged 4 to 7.

SGT-001 is a microdystrophin developed by Solid Biosciences that contains a neuronal nitric oxide synthase binding (nNOS) domain responsible for protection against ischemia-induced muscle injury by maintaining nitric oxide (NO) signaling at the muscle sarcolemma. SGT-001 is currently being evaluated in a phase 1/2 study called IGNITE DMD (NCT03368742). 91 Interim analysis of 2 years (n = 9 boys) demonstrated common drug-related laboratory abnormalities (thrombocytopenia, anemia), and serious adverse events: systemic inflammatory response syndrome (n = 2), thrombocytopenia (n = 1), and immune hepatitis (n = 1), which were resolved. 91 Results showed durable microdystrophin expression and localization on nNOS to the membrane in biopsies collected at time points ranging from 12-24 months post-dosing. In addition, patients had stable Six Minutes Walking Test (6MWT) distances and NSAA scores compared to natural history, and improvements in Forced Vital Capacity (FVC) and Peak Expiratory Flow (PEF) compared to baseline and natural history. 91 Up to three years post-dosing, there was durability of treatment effect. Subjects receiving 2E14 vg/kg maintained motor and pulmonary functions compared to expected natural history declines. 92

Gene replacement therapy using microdystrophin is a major advance in the treatment of DMD. However, there are important safety concerns. Among the adverse events of therapy, an immune response against protein epitopes encoded by the microdystrophin construct can generate a cytotoxic response mediated by T cells in patients missing these epitopes, which may cause severe myositis and myocarditis. 93 This has been particularly observed in patients with mutations encompassing exons 8 to 21. Another important limitation of gene therapy for DMD is that as AAV vectors do not integrate into the muscle fiber genome and do not tend to infect satellite cells, with the process of muscle renewal and regeneration, the effectiveness of the therapy tends to be lost with time. 94

### Antisense oligonucleotides (ASOs) therapy for DMD

Antisense oligonucleotides (ASOs), or phosphorodiamidate morpholino oligomers, can induce therapeutic exon skipping during pre-mRNA processing to restore the reading frame of the primary transcript of *DMD* . 95 As a result, truncated but partially functional dystrophin is produced, potentially slowing down the disease progression and causing a milder Becker muscular dystrophy phenotype.

Eteplirsen is an ASO from Sarepta Therapeutics that binds to a complementary region in the *DMD* exon 51 pre-mRNA and causes its skipping during the mRNA splicing process. 96 Around 13–14% of DMD cases have mutations that can be potentially treated by skipping exon 51. 97 Eteplirsen received accelerated approval from the FDA in September 2016. In clinical trials, eteplirsen was shown to be safe, and capable of increasing dystrophin levels in muscle tissue and maintaining the motor function of DMD patients. 96 97 98 99 100

Golodirsen is an ASO from Sarepta Therapeutics approved by the FDA under accelerated review for the treatment of DMD in patients with exon 53 amendable skipping mutations (approximately 8% of all DMD mutations). 101 In clinical trials A significant increase in exon 53 skipping and dystrophin protein expression was demonstrated. 102 103 104 In addition, mild improvement in 6MWT, and a slowing decline of FVC occurred. 103

Viltolarsen is an ASO designed to treat DMD in patients with a confirmed mutation of the DMD gene amenable to exon 53 skipping. 105 106 Viltolarsen is approved in the US by the FDA and in Japan. An increase of dystrophin value of 5.9% was observed, 105 and viltolarsen-treated patients presented stabilization of motor function over 4 years of extension study. 106

### Readthrough therapies for DMD

DMD is caused by a nonsense mutation in the *DMD* in 10–15% of cases. Nonsense mutations result in the production of truncated, non-functional dystrophin. Ataluren (Translarna®) is an oral treatment designed to promote the synthesis of full-length dystrophin through ribosomal readthrough of an in-frame premature stop codon caused by a nonsense mutation in the dystrophin mRNA. 107

A randomized, double-blind, placebo-controlled study, including males ≥ 5 years with DMD, assessed the safety and efficacy of ataluren (n = 57) or placebo (n = 57). The primary endpoint favored ataluren 10, 10, 20 mg/kg versus placebo. 108 A phase 3, randomized, double-blind, placebo-controlled trial, included 228 outpatient boys with DMD aged 7 to 16 years. 109 The study showed that change in 6MWD did not differ significantly between patients in the ataluren group and those in the placebo group. 109 However, a significant effect of ataluren in the prespecified subgroup of patients with a baseline 6MWD of 300 meters or more to less than 400 meters was noted. 109 However, to date, long-term follow-up on study participants (treated and untreated with atalurem) has not been reported.

The safety and effectiveness of ataluren in patients with nonsense mutation DMD in the STRIDE registry (real-world treatment with ataluren) were compared with the CINRG Duchenne Natural History Study. 110 Ataluren treatment significantly delayed age at loss of ambulation by 4 years and age at decline to predicted FVCof < 60% and < 50% by 1.8 years and 2.3 years, respectively. 110 However, the major limitation of this study is the fact that the comparator control group included DMD patients with different types of mutations, not just nonsense mutations, which can certainly affect the interpretation of the results. 110

Ataluren is not approved by the FDA. The Human Medicines Committee (CHMP) of the European Medicines Agency (EMA) gave conditional marketing authorization in 2014. However, in October 2023, in a preliminary analysis, the committee concluded that Ataluren's benefit-risk balance is negative and therefore recommended not renewing the marketing authorization in Europe. 111 In Brazil, ANVISA approved ataluren in 2019 and recently expanded the age range for its use from 2 years onwards.
